# Transcriptomic Analysis of Flowering Time Genes in Cultivated Chickpea and Wild *Cicer*

**DOI:** 10.3390/ijms24032692

**Published:** 2023-01-31

**Authors:** Maria Gretsova, Svetlana Surkova, Alexander Kanapin, Anastasia Samsonova, Maria Logacheva, Andrey Shcherbakov, Anton Logachev, Mikhail Bankin, Sergey Nuzhdin, Maria Samsonova

**Affiliations:** 1 Mathematical Biology and Bioinformatics Laboratory, Peter the Great St. Petersburg Polytechnic University, 195251 St. Petersburg, Russia; 2 Centre for Computational Biology, Peter the Great St. Petersburg Polytechnic University, 195251 St. Petersburg, Russia; 3 Center of Life Sciences, Skolkovo Institute of Science and Technology, 121205 Moscow, Russia; 4 Laboratory of Microbial Technology, All-Russia Research Institute for Agricultural Microbiology, 196608 St. Petersburg, Russia; 5 Section of Molecular and Computational Biology, University of Southern California, Los Angeles, CA 90089, USA

**Keywords:** flowering time genes, cultivated chickpea, wild *Cicer*, transcriptome sequencing, differential gene expression, vernalization response

## Abstract

Chickpea (*Cicer arietinum* L.) is a major grain legume and a good source of plant-based protein. However, comprehensive knowledge of flowering time control in *Cicer* is lacking. In this study, we acquire high-throughput transcriptome sequencing data and analyze changes in gene expression during floral transition in the early flowering cultivar ICCV 96029, later flowering *C. arietinum* accessions, and two wild species, *C. reticulatum* and *C. echinospermum*. We identify *Cicer* orthologs of *A. thaliana* flowering time genes and analyze differential expression of 278 genes between four species/accessions, three tissue types, and two conditions. Our results show that the differences in gene expression between ICCV 96029 and other cultivated chickpea accessions are vernalization-dependent. In addition, we highlight the role of *FTa3*, an ortholog of *FLOWERING LOCUS T* in *Arabidopsis*, in the vernalization response of cultivated chickpea. A common set of differentially expressed genes was found for all comparisons between wild species and cultivars. The direction of expression change for different copies of the *FT-INTERACTING PROTEIN 1* gene was variable in different comparisons, which suggests complex mechanisms of FT protein transport. Our study makes a contribution to the understanding of flowering time control in *Cicer*, and can provide genetic strategies to further improve this important agronomic trait.

## 1. Introduction

The transition from the vegetative to the reproductive phase is a major developmental switch in flowering plants. Flowering time control plays a key role in domestication and crop productivity, and is regulated by multiple endogenous signals and environmental conditions [1,2,3]. Chickpea (*Cicer arietinum* L.) is a major grain legume and good source of plant-based protein [4,5]. However, the genetic mechanisms of flowering time regulation in *Cicer* remain far from understood.

**Figure 1:**
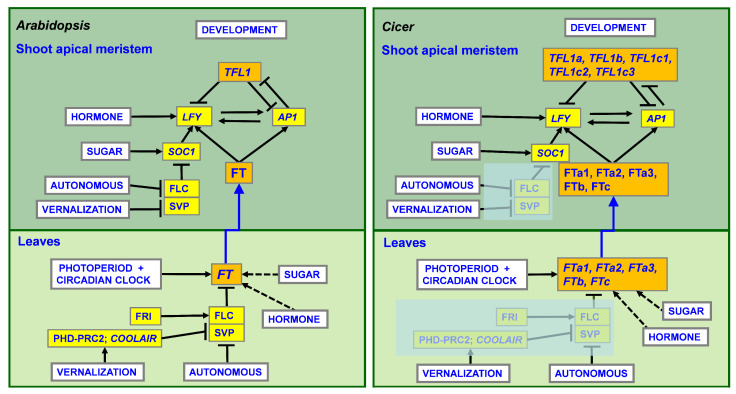
A general overview of the pathways involved in flowering initiation in *Arabidopsis*; the presumptive flowering time network in *Cicer*. *FT* and *TFL1* integrators, which have several copies in the *Cicer* genome, are highlighted in orange. Dashed arrows correspond to indirect/putative mechanisms (FLOR-ID database, [6]). The vernalization response mechanism via regulation of *FLC* gene, which is missing in *Cicer*, is shown in pale colors. The scheme shows only major regulators; for a more detailed description of flowering regulation in *Arabidopsis*, see [6,8].

Much of our current understanding of the genes involved in flowering time control is based on studies of the model species *Arabidopsis thaliana*. To date, more than 300 flowering time genes have been identified in *Arabidopsis*, including a number of key regulators. These genes are integrated into several major pathways [6] (Figure 1). The main signal responsible for floral promotion is encoded by the *FLOWERING LOCUS T (FT)* gene, the expression of which is induced in the leaves by numerous endogenous and exogenous signals [7]. These signals are encoded by genes from the ’photoperiod/circadian clock’, ’vernalization/ambient temperature’, ’autonomous’, ’hormone’, and ’sugar’ pathways [6,8,9] (Figure 1). Following the induction of *FT* gene expression, the FT protein moves from the leaves to the shoot apex where it activates meristem identity genes, including the major regulators *APETALA1 (AP1)* and *LEAFY (LFY)* [10,11,12]. These genes promote flowering by specification of the floral fate of shoot apical meristems, and act upstream of the floral organ identity genes. Unlike the FT protein, the *TERMINAL FLOWER1 (TFL1)* gene product functions as an ’anti-florigen’, and represses meristem identity genes [12,13,14].

Vernalization responsiveness in *Arabidopsis* depends on the regulation of the *FLOWERING LOCUS C (FLC)* gene, which encodes the MADS box transcription factor [15,16,17,18]. Before cold treatment, high levels of *FLC* transcription are provided by the FRIGIDA (FRI) complex [19]. The FLC protein represses the *FT* gene by binding to its first intron [20]. Vernalization induces *FLC* silencing through the action of many factors, including components of the ’autonomous’ and ’vernalization’ pathways, the PHD-PCR1 complex, and the *COOLAIR* complex [18,21,22] (Figure 1). This leads to de-repression of the *FT* gene and its activation by the ’photoperiod’ pathway, resulting in floral transition.

The most important gene in photoperiodic control of flowering in *Arabidopsis* is *CONSTANS (CO)*, which integrates signals from regulators such as *GIGANTEA (GI)*, *LATE ELONGATED HYPOCOTYL (LHY)*, *CRYPTOCHROME (CRY)*, *CRYPTOCHROME-INTERACTING BASIC-HELIX-LOOP-HELIX (CIB)*, *PHYTOCHROME (PHY)*, *CONSTITUTIVE PHOTOMORPHOGENIC 1 (COP1)*, *CYCLING DOF FACTORS (CDFs)*, and others [8,23,24,25].

Extensive research has revealed that the flowering pathways described in *A. thaliana* are largely conserved in legumes [26,27,28,29,30]. However, there are three main differences. First, genome evolution has led to many changes in the number of gene copies [31,32,33,34]. Legumes have multiple *FT* genes, which are organized in three subclades, (*FTa*, *FTb*, and *FTc*), as well as multiple *TFL1* genes [26,27,34,35,36]. This results in high complexity of the genetic networks involved in the activation of *FT* expression in the leaves and transmission of multiple FT and TFL1 signals to meristem identity genes [37,38] (Figure 1). Moreover, different *FT* and *TFL1* genes may possess distinct patterns of regulation with respect to environmental cues and tissue specificity [26,27]. Second, vernalization-sensitive legume species generally lack *FLC* orthologs, and the molecular mechanisms of vernalization response in these species are largely unknown [34,39,40] (Figure 1). However, as in *Arabidopsis*, *FT* genes appear to be major targets of vernalization in legumes [27,36,40]. Third, the *CO* gene presumably does not play a central role in photoperiodic regulation in legumes, as suggested by studies of *CO* homologues (*COL* genes) in *Medicago truncatula* and pea (*Pisum sativum*) [41,42,43].

In *Cicer*, multiple loci responsible for flowering time control have been discovered, including the *Early flowering* (*Efl1, Efl2, Efl3*) loci, a genomic region in the central portion of chromosome three and a “hot spot” on linkage group (LG) four [41,44,45,46,47,48,49,50]. Nevertheless, the expression and function of the genes underlying these loci remains under investigation. Recent studies have shown that *Efl1*, an ortholog of *Arabidopsis EARLY FLOWERING 3* (*ELF3*) [35], and a cluster of three *FT* genes (*FTa1-FTa2-FTc*) within the quantitative trait locus (QTL) *DTF3A* on chromosome three both play an important role in the early flowering of domesticated chickpea [50]. Overall, there are five *FT* genes in *Cicer*; however, they are differently distributed within subclades compared to pea and *Medicago* [50]. In total, there are three *FTa* genes (*FTa1*, *FTa2*, and *FTa3*), the *FTb* gene, and the *FTc* gene. In addition, there are five *TFL1* genes: *TFL1a*, *TFL1b*, *TFL1c1*, *TFL1c2*, and *TFL1c3* [50].

Regarding tissue specificity, a global transcriptome analysis study has characterized the gene expression in vegetative and reproductive tissues of domesticated *Cicer* [51]. However, similar analyses have not been performed for wild *Cicer* species. Recent research has characterized the transcriptome landscape of inflorescence development in chickpea and identified candidate regulators such as *ELF3a*. Results from this work suggest the importance of *LFY* and *AP1* regulation during inflorescence and floral development of *C. arietinum* [52].

In addition to *C. arietinum*, which is an annual cultivated chickpea, the genus *Cicer* includes both perennial and annual wild species. *C. reticulatum* and *C. echinospermum* are the annual wild species most closely related to the domesticated chickpea. *C. reticulatum* is the immediate progenitor of *C. arietinum*, while *C. echinospermum* meets the criteria for the secondary gene pool of *C. arietinum* [5,53]. Vernalization responsiveness is inherent to annual wild *Cicer* species, including *C. reticulatum* and *C. echinospermum*, but it is considered to be lost from the cultivated chickpea [54,55,56]. During domestication, *C. arietinum* has been transformed from a winter crop to a summer crop [50,57]; however, the sensitivity of cultivated chickpea to vernalization remains debatable. Although the vernalization response appears to be lost in many early flowering accessions, recent publications suggest that it may be preserved in the later-flowering varieties [56,58]. In this regard, the analysis of changes in gene expression in response to vernalization treatment in cultivated chickpea is of particular interest. A major QTL for the vernalization response in *Cicer* has been discovered on linkage group three (LG3) of the chickpea genetic map, in approximately the same genomic region as the *FTa1-FTa2-FTc* cluster [50]; however, the individual genes responsible for vernalization sensitivity have not yet been characterized [59].

In this study, we consider a high-throughput transcriptomic dataset obtained for the cultivated chickpea and two wild species, *C. reticulatum* and *C. echinospermum*, from leaves and floral buds after vernalization and without vernalization treatment. The elite early flowering cultivar ICCV 96029 [48,60] carries a number of mutations, which include an 11 bp deletion in the first exon of the *ELF3* gene [35]. ICCV 96029 was considered separately from the other *C. arietinum* accessions and referred to as ‘mutant’. Although ICCV 96029 has been reported to be photoperiod insensitive, it preserves the function of circadian clock genes [35]. We analyzed differential expression of 278 *Cicer* orthologues of *A. thaliana* flowering time genes [6] between tissue types, conditions, and species/accessions. The most attention was paid to the analysis of *FT* genes, their regulators, and their targets. We sought to address the following questions. (1) How does the expression of flowering time genes depend on tissue type? (2) Does the domesticated chickpea respond to vernalization? (3) What is the difference in gene expression between ICCV 96029 and other cultivated chickpea accessions? (4) How does the expression of flowering-related genes differ between wild and domesticated *Cicer*?

We believe that our results can improve knowledge of the genes involved in the regulation of flowering time in *Cicer* and in other legumes.

## 2. Results

### 2.1. Differential Gene Expression between Tissue Types

**Figure 2:**
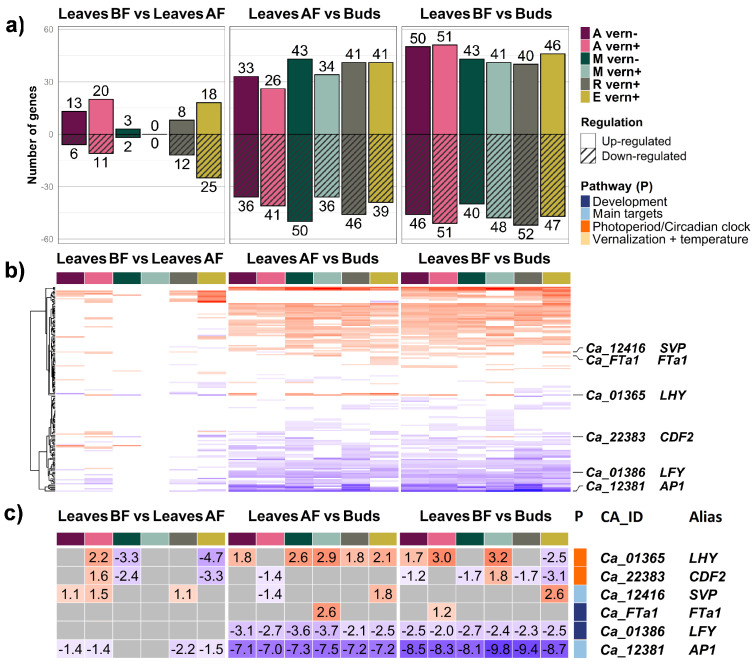
Differential expression of flowering time genes in comparisons between tissue types. (**a**) Number of flowering time genes differentially expressed between three tissue types. Each comparison was analyzed for the following species/accessions and conditions: ‘A’—*C. arietinum*, ‘M’—ICCV 96029 mutant, ‘R’—*C. reticulatum*, ‘E’—*C. echinospermum*, ‘vern+’—after vernalization, and ‘vern-’—without vernalization. (**b**) The direction of gene expression regulation did not change with respect to species/accessions or conditions. The rows of the heatmap visualize the values of the log2 fold change (FC) for the expression of all genes in the dataset between three tissue types. It is evident that the color in each row (corresponding to individual genes) does not change between the columns (corresponding to species/accessions and conditions) within each comparison. FC values for three genes, which do not follow this rule (the orthologs of *LHY*, *CDF2*, and *SVP*) are shown in panel (**c**). (**c**) The heatmap shows the FC values for differential expression of the genes listed in the right-hand panel. Up- and downregulation are shown in red and blue, respectively. Gray cells indicate the absence of differential expression between tissue types.

First, we analyzed the differences in gene expression between three tissue types: leaves before flowering initiation (leaves BF), leaves after flowering initiation (leaves AF), and the early buds. The number of differentially expressed genes (DEGs) between the leaves (at any stage) and the buds was twice that between leaves BF and leaves AF. We detected 66 DEGs between leaves BF and leaves AF, 128 DEGs between leaves AF and buds, and 147 DEGs between leaves BF and buds (Figure 2a).

A number of DEGs varied across species/accessions and conditions (columns, labeled with different colors in Figure 2a). In the comparison between two types of leaf tissues (leaves BF and leaves AF), the highest number of DEGs was detected for *C. echinospermum* after vernalization (43 DEGs) and *C. arietinum* (31 DEG), while very few DEGs were found in mutant ICCV 96029, with five DEGs without vernalization and no DEGs after vernalization (Figure 2a). This variation was much lower in the comparison between leaves AF and flower buds, where we found the maximum number of DEGs in the mutant without vernalization (93 DEGs) and the minimum number in *C. arietinum* (69 and without vernalization and 67 DEGs after vernalization) (Figure 2a). The comparison between leaves BF and floral buds showed minimal variation, with a large number of DEGs in all species/accessions and conditions (Figure 2a).

Interestingly, the direction of regulation did not change with respect to species/ accession and condition when an individual DEG was up- or downregulated in the comparison between two tissue types (Figure 2b, Supplementary Tables S3–S5). Of all DEGs, only three genes did not follow this rule; *LHY* and *CDF2*, which in *Arabidopsis* encode transcription factors from the ’photoperiod/circadian clock’ pathway, changed the direction of regulation in two comparisons, leaves BF vs. leaves AF and leaves BF vs. buds (Figure 2b,c). The same was observed in the comparison between leaves AF and buds for the *SHORT VEGETATIVE PHASE (SVP)* gene (Figure 2b,c). This suggests that the tissue-specific expression of these genes is highly dependent on species/accession and condition.

The expression of the *FTa1* gene was upregulated in leaves AF of mutant ICCV 96029 after vernalization and in leaves BF of *C. arietinum* after vernalization when compared with buds (Figure 2c, Supplementary Table S3). An ortholog of the *Arabidopsis AP1* gene showed the most dramatic difference in gene expression between leaves and buds. The log2 fold change values (FC) in the comparisons of buds with leaves AF and leaves BF were 7.0–7.5 and 8.1–9.9, respectively (Figure 2c, Supplementary Table S3). The expression of another meristem identity gene *LFY*, acting in synergy with *AP1*, was upregulated in the buds, though with lower FC values (Figure 2c).

### 2.2. Vernalization Response in Cultivated Chickpea

We sought to check the vernalization responsiveness of the early flowering mutant ICCV 96029 in comparison with other *C. arietinum* accessions.

**Figure 3:**
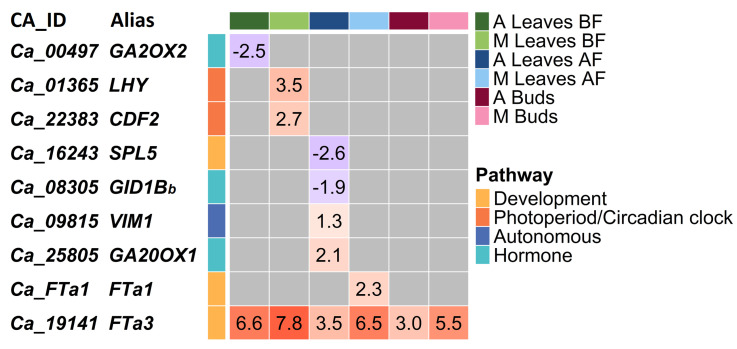
Changes in gene expression in cultivated chickpea in response to vernalization. Each comparison was analyzed in three tissue types (leaves BF, leaves AF and buds) and in the following species/accessions: ‘A’—*C. arietinum*, ‘M’—ICCV 96029 mutant, ‘R’—*C. reticulatum*, and ‘E’—*C. echinospermum*. The heatmap shows the FC values for differential expression of the genes listed in the right-hand panel. Up- and downregulation are shown in red and blue, respectively. Gray cells indicate the absence of differential expression between tissue types. *FTa3* is upregulated by vernalization in both *C. arietinum* and mutant ICCV 96029.

As expected, in cultivated chickpea the number of DEGs between the two conditions (with and without vernalization) was very small, ranging from one to five genes depending on the tissue studied. All the DEGs are presented in Figure 3.

The ortholog of the *Arabidopsis FTa3* gene (*Ca_19141*) was upregulated by vernalization in all tissue types of both *C. arietinum* and ICCV 96029 (Figure 3). *FTa3* upregulation was higher in the mutant ICCV 96029 compared to other *C. arietinum* accessions; FC values ranged from 3.0 to 6.6 in *C. arietinum* and from 5.5 to 7.8 in the mutant. Maximum levels of *FTa3* activation were detected in leaves BF for both types of chickpea accessions.

In addition to *FTa3*, all DEGs in both mutant and other *C. arietinum* accessions were found in two tissue types, namely, leaves BF and leaves AF (Figure 3). In the mutant, all DEGs were upregulated by vernalization, including *LHY* and *CDF2* as well as another *FT* family member, *FTa1* (Figure 3).

In *C. arietinum*, five genes were differentially expressed in response to vernalization treatment; three were from the ’hormone’ pathway (encoding gibberellin oxydases *GA2OX2* and *GA20OX1* and the gibberellin receptor *GID1B*), one was the DNA methylation factor *VARIANT IN METHYLATION 1 (VIM1)*, and the last was the *SQUAMOSA PROMOTER BINDING PROTEIN-LIKE 5 (SPL5)* gene, which encodes an ortholog of transcription factor involved in the regulation of *FT*, *LFY*, and *AP1* in *Arabidopsis*. The expression of *SPL5* and two genes from the ’hormone’ pathway was downregulated in response to vernalization, while *GA20OX1* and *VIM1* were upregulated (Figure 3).

### 2.3. Flowering Time Genes That Differentiate ICCV 96029 from Other Cultivated Accessions

The early flowering ICCV 96029 mutant was reported to be photoperiod-insensitive due to a mutation in the chickpea homologue of the major *Arabidopsis* circadian clock gene *ELF3*. This mutation provides early induction and increased expression of *FT* genes while maintaining the rhythms and expression levels of the circadian clock genes [35]. Thus, it was interesting to compare gene expression between ICCV 96029 and other *C. arietinum* accessions in various tissue types, with particular attention to the expression and regulation of the photoperiod/circadian clock and *FT* genes.

**Figure 4:**
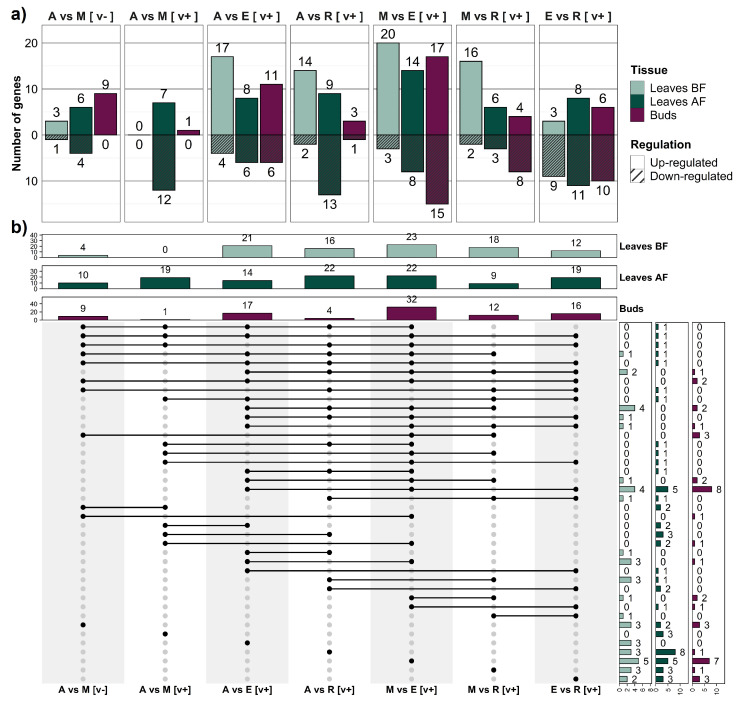
The number of flowering time genes differentially expressed between *Cicer* species/accessions. (**a**) The barplot shows the number of genes in different comparisons (top panel), where ‘A’—*C. arietinum*, ‘M’—ICCV 96029 mutant, ‘R’ – *C. reticulatum*, ‘E’—*C. echinospermum*), ‘vern+’—after vernalization, ‘vern-’—without vernalization. Each comparison was analyzed in three tissue types: leaves BF, leaves AF, and buds. (**b**) The UpSet plot shows the number of genes differentially expressed in several comparisons simultaneously. Each column in the panel matrix corresponds to the comparison between the specified species/accessions; the rows represent all possible intersections of these comparisons (unique and overlapping DEGs). The panel matrix consists of filled and empty circles. Connected filled circles in a row indicate comparisons that are included in the intersection, while empty circles indicate that these comparisons are excluded from the intersection. If a circle is not connected with other circles, this comparison does not intersect with others. Three barplots in the top panel show the total number of genes differentially expressed between species/accessions in three tissue types. The three barplots at the right-hand side summarize the number of DEGs for each type of intersection.

**Figure 5:**
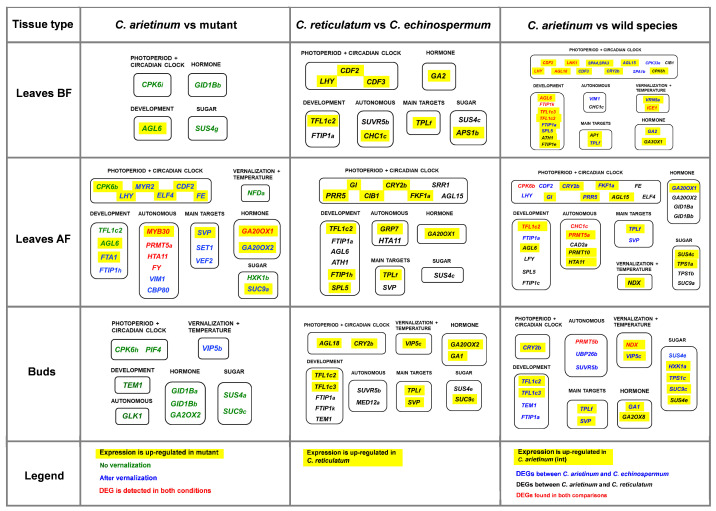
Flowering time genes differentially expressed in three comparisons between species/accessions: (1) *C. arietinum* vs. ICCV 96029 mutant; (2) *C. reticulatum* vs. *C. echinospermum*; (3) *C. arietinum* vs. two wild species (*C. reticulatum* and *C. echinospermum*). Each box represents the developmental pathway (see Figure 1). Gene upregulation in the particular species/accessions is shown by yellow shading of the gene’s name. Different comparisons are indicated by the text color (see legend at the bottom of the Figure).

The number of DEGs between ICCV 96029 and other *C. arietinum* cultivars was relatively small (Figure 4a). The maximum number of DEGs was detected in leaves AF, suggesting that this tissue is critical with respect to the differences between the two types of chickpea accessions (Figure 5). The expression of all genes from the ‘photoperiod/circadian clock’ pathway in this tissue type was upregulated in the mutant. We found differentially expressed orthologs of the *Arabidopsis* photoperiodic genes *CALCIUM DEPENDENT PROTEIN KINASE 6 (CPK6)* (FC = 1), *CDF2* (FC = 1.9), *MYB-RELATED PROTEIN 2 (MYR2)* (FC=1.4), and *FE* (FC = 1.1), as well as the circadian clock genes *LHY* (FC = 2.5) and *ELF4* (FC = 3.23) (Figure 5). In terms of condition specificity, nineteen DEGs were revealed in the leaves AF after vernalization treatment (shown in blue) and in ten DEGs without vernalization (shown in green) (Figure 5). Genes differentially expressed in specific conditions included *FTa1* (upregulated in ICCV 96029 after vernalization treatment, FC = 2.9) and *TFL1c2* (upregulated in *C. arietinum* without vernalization, FC = 2.1). Five genes, mostly belonging to the ‘autonomous’ pathway, were expressed differentially in both conditions (shown in red) (Figure 5).

A significantly smaller number of DEGs was found in tissues other than leaves AF (Figure 5). No DEGs were revealed in the leaves BF collected from vernalized plants (Figure 4a), and only four DEGs were detected in the same tissue type without vernalization. The expression of all these genes except for *AGAMOUS-LIKE 6 (AGL6)* was downregulated in the mutant ICCV 96029. In the flowering buds, the expression of all DEGs was downregulated in the mutant as well. In the buds of vernalized plants, only one gene, *VERNALIZATION INDEPENDENCE 5 (VIP5)* from the ‘Vernalization/temperature’ pathway, FC = 1.1, was differentially expressed, while in the buds of non-vernalized plants nine DEGs, including two photoperiod/circadian clock genes (*PHYTOCHROME INTERACTING FACTOR 4 (PIF4)* (FC = 1.2) and *CPK6* (FC = 1.3)), were found.

Our data showed that the differential expression between ICCV 96029 and other *C. arietinum* accessions was vernalization-sensitive (Figure 4a). For example, of 38 DEGs detected in all tissue types (Figure 5), only five were differentially expressed in both conditions. The remaining 33 genes were differentially expressed in specific conditions, specifically, 18 genes in plants without vernalization and 15 genes in vernalized plants (Figure 5). Remarkably, out of 18 DEGs found in plants without vernalization, only three genes were upregulated in the mutant. On the contrary, after vernalization, we found that nine genes were upregulated in the mutant, while six genes were upregulated in other *C. arietinum* accessions (Figure 5). Thus, the number of genes upregulated in ICCV 96029 increased significantly after vernalization treatment.

### 2.4. Differences in Flowering Time Gene Expression between *C. reticulatum* and *C. echinospermum*

The number of DEGs between these two wild species was twice that between two types of cultivars under the same conditions (after vernalization treatment) (Figure 4a). This reflects the evolutionary distance between *C. reticulatum* and *C. echinospermum*. Interestingly, most genes were upregulated in *C. reticulatum* compared to *C. echinospermum* (Figure 4a and Figure 5).

The number of DEGs from the ‘photoperiod/circadian clock’ pathway was highest in the leaves AF (Figure 5).

We did not reveal any difference in expression of the *FT* gene orthologs between *C. reticulatum* and *C. echinospermum*. Interestingly, the orthologs of the *Arabidopsis FT-INTERACTING PROTEIN 1 (FTIP1)* gene, which encodes an endoplasmic reticulum protein involved in the transport of the *FT* gene product [61], were differently expressed between the two wild species: *FTIP1a* (Ca_00020) and *FTIP1*κ (Ca_19710) were upregulated in *C. echinospermum*, while *FTIP1h* (Ca_14215) was upregulated in *C. reticulatum* (Supplementary Table S1, Figure 5). This points to the complex mechanisms of FT protein transport, which can have distinct features in each species.

Two orthologs of the *Arabidopsis TFL1* gene were upregulated in *C. reticulatum* (Figure 5). It is noteworthy that *TFL1c2* expression was upregulated in all tissue types (FC = 2.5–3.3), while *TFL1c3* showed upregulation only in the buds (FC = 5.7). This suggests stronger negative control of flowering promotion in *C. reticulatum* compared to *C. echinospermum*.

A number of genes were differentially expressed in two or three tissue types, which suggests their major role in flowering time variation between wild species. The *Arabidopsis TOPLESS (TPL)* gene ortholog (*TPLf, Ca_14764*) was upregulated in *C. reticulatum* in all tissue types, while the ortholog of the *CRY2* gene (*CRY2b, Ca_23164*) was upregulated in *C. reticulatum* in leaves AF and buds (Figure 5). On the contrary, the ortholog of the *SVP* gene had a tissue-specific mode of regulation; it was upregulated in the leaves AF and downregulated in the flower buds of *C. reticulatum*. The expression of the *SUCROSE SYNTHASE 4 (SUS4)* gene ortholog from the ‘Sugar’ pathway (*SUS4c, Ca_03475*) was upregulated in *C. echinospermum* in all tissue types (FC = 4.2 in leaves BF, FC = 4.6 in the leaves AF, and FC = 3.2 in the buds) (Figure 5, Supplementary Tables S1 and S6–S8).

### 2.5. Comparison of Gene Expression between Wild and Cultivated *Cicer*

**Figure A1:**
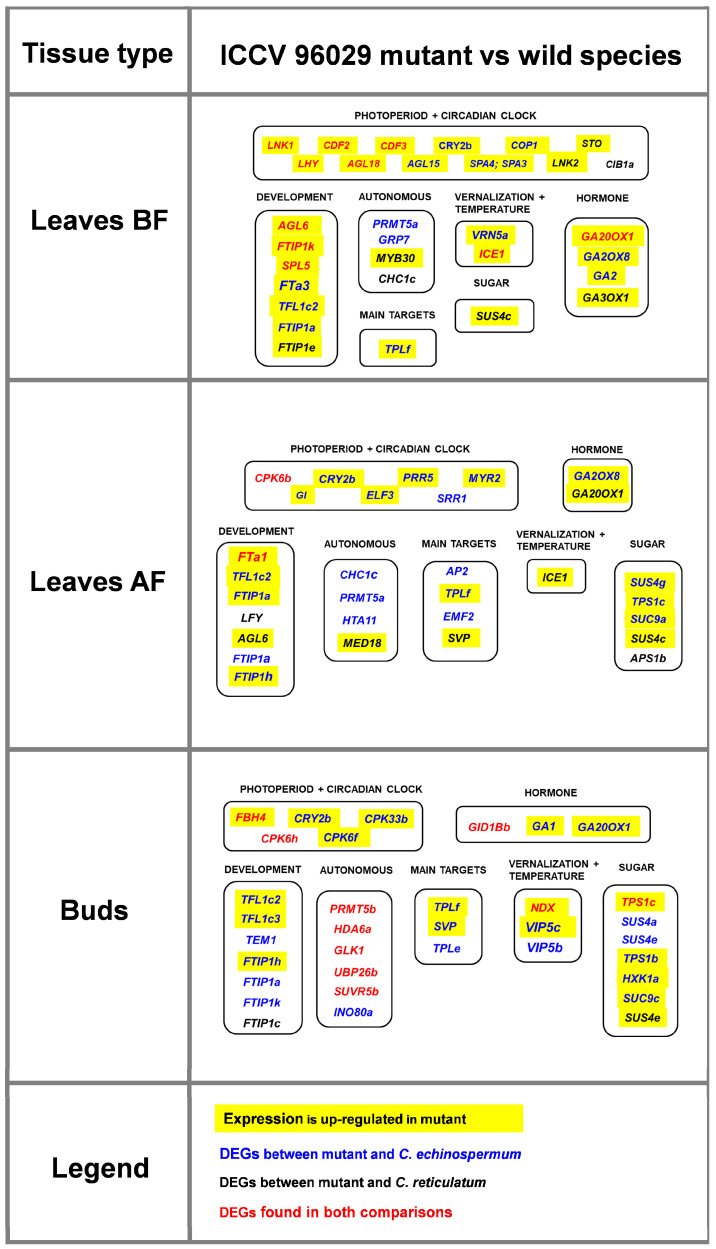
Flowering time genes differentially expressed between mutant ICCV 96029 and two wild species (*C. reticulatum* and *C. echinospermum*). Each box represents the developmental pathway (See Figure 1). Gene upregulation in a particular species/accession is shown by yellow shading of the gene name. Different comparisons are indicated by text color (see legend at the bottom of the figure).

The largest number of differently expressed genes from the ‘photoperiod/circadian clock’ pathway between wild and cultivated *Cicer* was detected in the leaves BF (Figure 5 and Figure A1). This could be explained by the early induction of light-signaling genes in cultivated chickpea compared to wild *Cicer*. These genes were mostly upregulated in cultivated chickpea. The number of ‘photoperiod/circadian clock’ DEGs decreased in the leaves AF, and was the lowest in flowering buds. On the contrary, in the buds we found that an increased number of DEGs from the ‘sugar’ pathway were found (Figure 5 and Figure A1). This trend was evident when comparing both types of cultivated *Cicer* with wild species.

The expression of *FT* genes was upregulated in the ICCV 96029 when contrasted with wild species. The difference in *FTa3* gene expression in leaves BF was only significant when the mutant was compared with *C. echinospermum*, while the difference in *FTa1* gene expression in leaves AF was significant when comparing the mutant with both wild species. The orthologs of the *Arabidopsis FTIP1* gene were differently expressed in all comparisons between wild and cultivated *Cicer* and in all tissue types. As was the case in the comparison between wild species, the direction of regulation varied between different copies of the *FTIP* gene (Figure 5 and Figure A1).

The expression of the *TFL1c* ortholog was upregulated in cultivated chickpea, pointing to the higher level of repression of its targets, namely, *LFY* and *AP1* (Figure 5 and Figure A1). Indeed, *LFY* was downregulated in both mutant and *C. arietinum* in comparison with *C. reticulatum* in the leaves AF (shown in black in Figure A1). Because the *FT* genes perform flowering induction via activation of meristem identity genes, different directions of regulation of *FT* and *LFY* could underlie variations in the activator/repressor balance required for floral promotion in wild and cultivated *Cicer*.

The DEGs found in most tissue types were largely consistent with those detected in the comparison between the two wild species. The orthologs of the *TPL* and *CRY2* genes (*TPLf*) and *CRY2b*) were upregulated in cultivated chickpea compared with *C. echinospermum* in all tissue types. The *SVP* gene was upregulated in the buds in cultivated chickpea accessions and leaves AF of the ICCV 96029 mutant and downregulated in leaves AF of *C. arietinum*, while multiple copies of the *SUS4* gene showed either up- or downregulation in cultivated chickpea in comparison with wild *Cicer* (Figure 5 and Figure A1, Supplementary Tables S6–S8).

### 2.6. Verification of Transcriptomic Data by Quantitative Real-Time PCR Assay

To validate the differential expression results obtained by RNAseq assay, we analyzed expression of four genes (*FTa1, LFY, LHY,* and *TFL1c2*) by qPCR in two random samples from our dataset (Figure A3). Both samples were leaves AF of *C. arietinum*: #32 after vernalization, and #38 without vernalization. The results of qPCR for three technical replicates are shown in Figure A3b.

We estimated differences in gene expression between the two samples by two methods, then compared FC values obtained by RNAseq and qPCR. The qPCR results showed the same direction of gene expression regulation as RNAseq (Figure A3a), confirming the reliability of RNAseq analysis.

## 3. Discussion

### 3.1. The Defining Role of Tissue Type in Differential Expression of Individual Genes

In the between-tissue comparison, the direction of regulation of an individual gene was gene-specific and did not change with species/accession or condition (Figure 2b). This highlighted the leading role of tissue type in defining the difference in expression of individual genes.

However, three exceptions were found: the direction of expression regulation of *LHY, CDF2*, and *SVP* genes varied between species/accessions and conditions (Figure 2b,c), which may be explained by the function of these genes. In *Arabidopsis*, *LHY* encodes a transcription factor that plays a major role in circadian clock regulation, while *CDF2* regulates blue light signaling and miRNA biogenesis [62] and is putatively regulated by *LHY* [63]. The *SVP* gene is a major regulator controlling the effect of environmental cues in floral induction [64]. It is likely that in *Cicer* these genes are involved in environmental signal processing as well, necessitating the need to tune gene expression in response to external and internal cues.

Our results clearly indicate a larger difference in gene expression between leaves and floral buds than between that of two types of leaves. This observation is consistent with a previous study suggesting the existence of different transcriptional programs in chickpea vegetative and flower tissues [51].

With regard to major flowering regulators, we detected that *FTa1* expression was upregulated in leaf tissues compared to buds in cultivated chickpea after vernalization (Figure 2 and Supplementary Tables S4 and S5). This is consistent with earlier published results [50]; however, it indicates a possible dependence of tissue-specific *FTa1* expression on vernalization treatment.

On the contrary, the expression of meristem identity genes *AP1* and *LFY* was upregulated in the buds (Figure 2c). In *Arabidopsis*, *LFY* is expressed in the inflorescence and floral meristems and activates the expression of *AP1* and floral organ identity genes [10,65,66,67]. The expression of both genes is generally conserved in legumes [68,69], although with a few functional differences. For example, the pea homologue of *LFY* is involved in the regulation of complex leaf development along with its role in the initiation of floral meristems [67,68,70]. Upregulation of *LFY* in the flower buds of all *Cicer* species/accessions does not suggest any function of this gene in the leaf tissue.

### 3.2. The FTa3 Gene Is Upregulated by Vernalization in Cultivated Chickpea

During the process of domestication, chickpea was transformed from an autumn-sown crop to a spring-sown crop, providing maturation during summer and avoiding Ascochyta blight disease in winter. Due to these breeding attempts, the vernalization responsiveness is considered to have been lost from the domesticated chickpea, in contrast to its wild relatives [54,55,56]. Interestingly, the analysis of different cultivated accessions revealed that the late-maturating varieties continue to respond to vernalization, unlike the early-maturating varieties [56].

**Figure 6:**
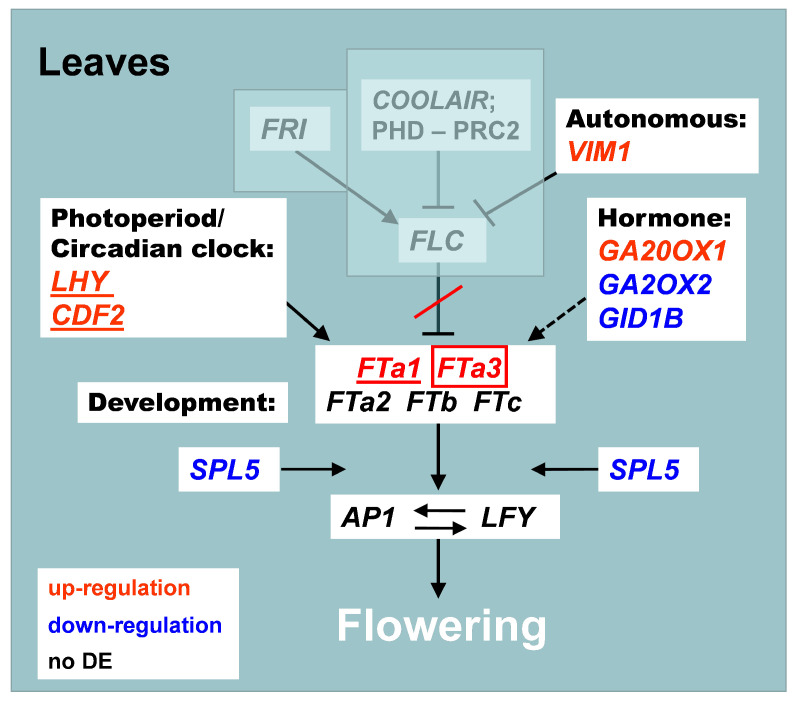
Putative regulatory interactions underlying flowering promotion in response to vernalization in chickpea. The scheme places the DEGs from Figure 3 into the context of Figure 1. The names of the DEGs found in the mutant ICCV 96029 are underlined. The *FTa3* gene, which is the common DEG in mutant and other *C. arietinum* accessions, is shown in the box. Upregulated and downregulated genes are shown in red and blue, respectively. The dashed arrow corresponds to the indirect/putative mechanism (FLOR-ID database, [6]). The mechanism of vernalization response via *FLC*, which is presumably missing in *Cicer*, is shown in pale colors.

In our analysis, we found that the number of DEGs was very small in both the early-flowering ICCV 96029 mutant and the later-maturating *C. arietinum* accessions (Figure 3). Nevertheless, DEGs in the mutant and *C. arietinum* generally belonged to different regulatory pathways, with an exception for the ’Development’ pathway (Figure 6).

Remarkably, all DEGs in the early-maturating mutant were upregulated by vernalization. They included *LHY* and *CDF2* from the ’Photoperiod/circadian clock’ pathway and two *FT* genes, *FTa1* and *FTa3*. *CDF2* activation by vernalization treatment is non-trivial, as in *Arabidopsis* it represses *FT* transcription [71].

On the contrary, vernalization treatment in *C. arietinum* affected the expression of three genes from the ’hormone’ pathway, *VIM1* from the ’autonomous’ pathway, and two genes from the ’development’ pathway, *FTa3* and *SPL5* (Figure 6). In *Arabidopsis*, the *SPL5* gene is regulated by *FT* and activates expression of *AP1* [72,73]. It has been recently reported that *SPL5* is involved in the timing of cold-induced floral transition in rapeseed (*Brassica napus*) [74]. Downregulation of *SPL5* by vernalization in *C. arietinum* presumably underlies the timing of the vernalization response in *Cicer*. In *Arabidopsis*, VIM proteins regulate epigenetic silencing via histone modification and DNA methylation [75]. In *C. arietinum*, upregulation of the *VIM1* ortholog may contribute to silencing of the unknown *FT* repressor, thereby promoting activation of the *FT* genes by vernalization.

Interestingly, in both mutant and *C. arietinum*, the *FTa3* gene was up-regulated by vernalization in all tissue types (Figure 3 and Figure 6). Moreover, the *FTa3* FC values were higher in the early flowering mutant compared with the other chickpea accessions, which is not consistent with the previously reported vernalization insensitivity of the early flowering cultivars [56].

The data on narrow-leafed lupin *L. angustifolius* and *Medicago trancatula* suggest that the *FT* family genes may be the main targets of vernalization in legumes [27,36]. However, unlike the vernalization-sensitive genes from the *FTa1* subclade, the representatives of the *FTa3* subclade, namely, *MtFTa3*, *LanFTa1*, and *LanFTa2*, were not involved in vernalization response [36]. Further studies of wild and cultivated accessions are required in order to decipher the role of the *FTa3* gene in vernalization-induced flowering in *Cicer*.

### 3.3. Differential Gene Expression between ICCV 96029 and Other Cultivated Accessions Depends on Vernalization Treatment

According to the previous results, the early flowering ICCV 96029 mutant is photoperiod-insensitive, although with an apparently preserved function of the circadian clock genes [35]. Thus, a difference could be expected in the expression of light-signaling genes in the mutant compared to the later flowering *C. arietinum* accessions.

The maximum number of DEGs was detected in the leaves AF, suggesting that this tissue type is critical with respect to the difference in gene expression between the mutant and *C. arietinum*. In the leaves AF, all genes from the ’photoperiod/circadian clock’ pathway were upregulated in ICCV 96029 (Figure 5), as was with the ortholog of the *FTa1* gene, which plays a major role in the promotion of early flowering in *M. trancatula* and *P. sativum* [26,27,76,77]. The same upregulation was detected for the ortholog of the *AGL6* gene, encoding an important positive regulator of flowering [78]. On the contrary, the expression of the ’anti-florigen’ *TFL1c2* was downregulated in the mutant, which is consistent with the early maturation of this cultivar and confirms previous results [35,79].

Remarkably, most genes showed differential expression between ICCV 96029 and other *C. arietinum* accessions in a specific condition. For example, in the mutant, *FTa1* and *AGL6* were upregulated in the vernalized leaves, while *TFL1c2* was downregulated in the leaves AF without vernalization (Figure 5). Only five DEGs, mostly belonging to the ‘autonomous’ pathway, were expressed differentially in both conditions (Figure 5).

The number of genes differentially expressed between ICCV 96029 and other *C. arietinum* cultivars depended on vernalization treatment. We found a threefold increase in the number of genes activated in the early flowering mutant after vernalization compared to the nonvernalized data (Figure 4a). This suggests a role for vernalization in gene expression differences between early and later flowering *C. arietinum* accessions.

### 3.4. Shared DEGs in the Comparison between Two Wild Species and between Cultivated and Wild *Cicer*

We found many shared DEGs in our comparisons of two wild species and wild species with cultivated *Cicer* accessions (Figure 4b and Figure 5). For example, a copy of the *TPL* ortholog (*TPLf*) was upregulated in cultivated chickpea, in contrast with wild species, as well as in *C. reticulatum* as compared to *C. echinospermum* (Figure 5 and Figure A1). In *Arabidopsis*, the TPL co-repressor is involved in modulation of gene expression in diverse developmental processes, including photoperiodic flowering [80,81]. The same pattern of regulation was inherent to orthologs of the *TFL1* gene in all comparisons, and to another major repressor, *SVP*, in the buds (Figure 5). In the leaves AF, *SVP* was upregulated in *C. echinospermum* as compared with both *C. reticulatum* and *C. arietinum*.

**Figure 7:**
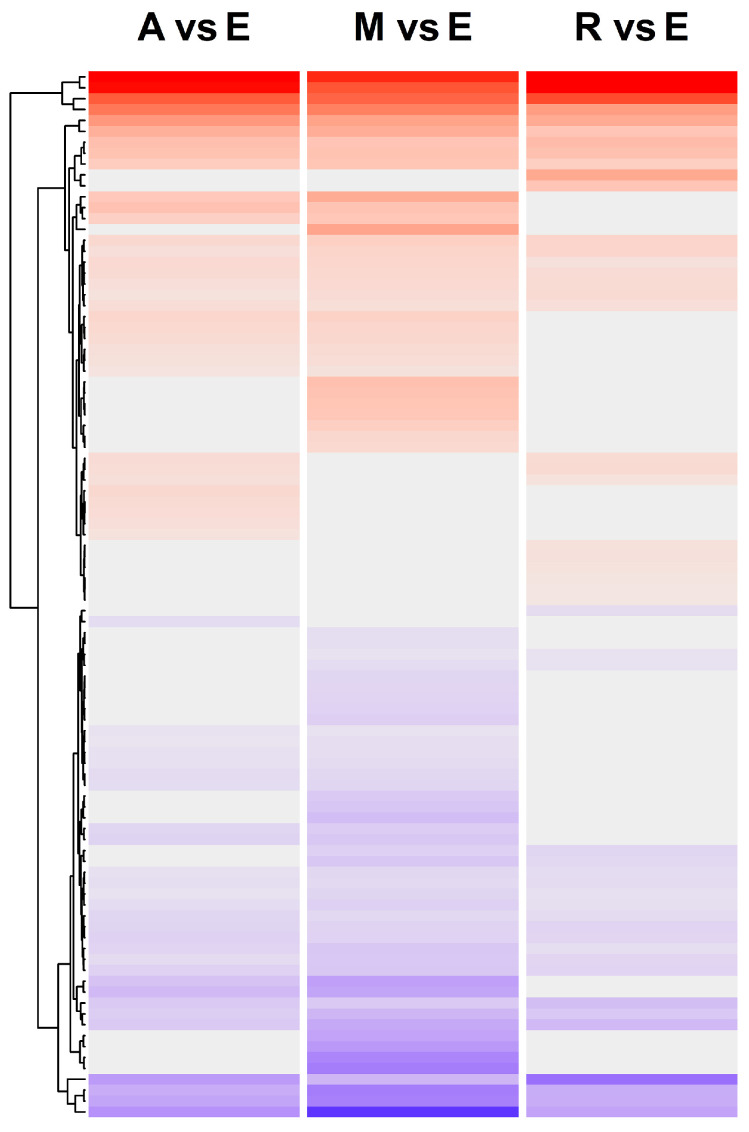
Comparisons between *C. echinospermum* and *C. reticulatum* and between *C. echinospermum* and cultivated *Cicer* share the same direction of expression regulation. The heatmap displays FC values for the comparisons of *C. arietinum* (A), the ICCV 96029 mutant (M), and *C. reticulatum* (R) with *C. echinospermum* (E) in the early flower buds. It is evident that the color in each row (corresponding to an individual gene) does not change between the columns (corresponding to each of the comparisons). Up- and downregulation arw shown in red and blue, respectively. Gray cells correspond to an absence of differential expression between species/accessions. In the entire dataset, only two outliers were found, *Ca_26523 (PRMT10)* and *Ca_01826 (PRMT5a)*, both in the leaves AF (Figure A2).

**Figure A2:**
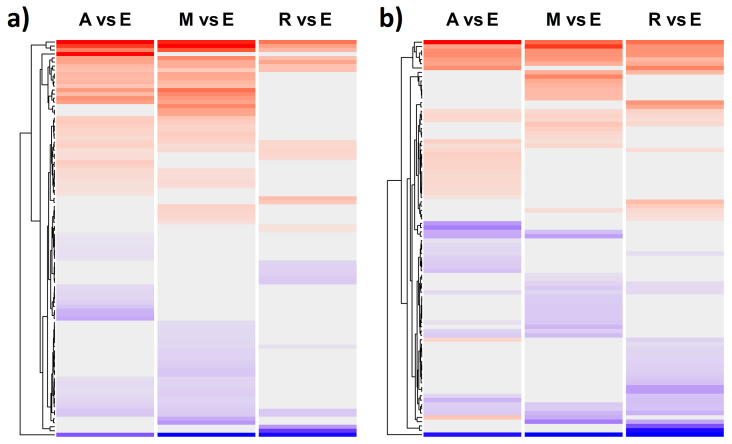
Comparisons between *C. echinospermum* and *C. reticulatum* and between *C. echinospermum* and cultivated *Cicer* share the same direction of expression regulation. The heatmap displays FC values for the comparisons of *C. arietinum* (A), ICCV 96029 mutant (M), and *C. reticulatum* (R) with *C. echinospermum* (E) in leaves BF (**a**) and leaves AF (**b**). It is evident that the color in each row (corresponding to individual genes) does not change between the columns (corresponding to each of the comparisons). Up- and downregulation are shown in red and blue, respectively. Gray cells correspond to an absence of differential expression between species/accessions. In of the entire dataset, only two outliers were found: *Ca_26523* (*PRMT10*) and *Ca_01826* (*PRMT5a*), both in the leaves AF.

Interestingly, our results showed that almost all genes had the same direction of expression regulation in comparisons of *C. echinospermum* with *C. reticulatum* and *C. echinospermum* with cultivated *Cicer* (Figure 7 and Figure A2). This suggests similarity in the mechanisms of flowering time regulation in *C. reticulatum* and cultivated chickpea, which is hardly surprising, as this species is regarded as the wild progenitor of domesticated varieties.

Considering that the *C. arietinum* varieties flower earlier than wild species even after vernalization treatment [56], the elevated levels of the flowering time repressors (*TPL*, *TFL1*, and *SVP*) in cultivated chickpea are unusual, and require a detailed analysis of the activator/repressor balance during flowering transition in different *Cicer* species.

### 3.5. Differences in Expression of FT and FTIP1 Genes between Wild and Cultivated *Cicer* and between C. reticulatum and C. echinospermum

A recent study has revealed the major contribution of the *FT* cluster, located on the *Cicer* chromosome 3 and includes the *FTa1, FTa2*, and *FTc* genes, to the difference in flowering time between cultivated chickpea and *C. reticulatum* [50]. We found upregulated expression of *FTa1* and *FTa3* orthologs in the ICCV 96029 mutant compared to wild species, suggesting that these genes contribute to the early flowering of this cultivar. On the contrary, no differences in *FT* expression were found between the two wild species (Figure 5 and Figure A1). This suggests a minor variation in flowering time between *C. reticulatum* and *C. echinospermum* after vernalization treatment, and confirms the previous observation [56].

In our dataset, we revealed fourteen orthologs of the *Arabidopsis FTIP1* gene, which encodes the endoplasmic reticulum membrane protein required for FT protein transport. In *Arabidopsis*, *FTIP1* shares mRNA expression and subcellular localization with the *FT* gene [61]. It is likely that the large number of copies of the *FTIP1* gene is related to the extended number of *FT* genes in *Cicer* as compared to *Arabidopsis*.

Remarkably, different copies of the *FTIP1* gene had variable directions of regulation both when comparing cultivated chickpea with wild species and *C. reticulatum* with *C. echinospermum* (Figure 5 and Figure A1). The transport of FT proteins through the phloem to the shoot apex, where they transfer information on environmental signals to meristem identity genes, plays a key role in flowering induction in *Arabidopsis* and legumes [26,61]. In *Arabidopsis*, loss-of-function mutations in *FTIP1* result in delayed flowering under long days [61]. The variability in the direction of *FTIP1* regulation suggests complex mechanisms of FT transport, which differ between *Cicer* species.

## 4. Materials and Methods

### 4.1. Plant Materials and Sample Collection

Four *C. arietinum* accessions (ICCV 96029, ICC 16201, CDC Frontier, and Consul), two *C. reticulatum* accessions (Bari3 and Cudi 1022), and two *C. echinospermum* accessions (610380 and 610381) were grown under long days (16 h light: 8-h dark photoperiod) in a climatic chamber at +26 °C. All seeds were vernalized at +4 °C in the dark for 30 days. For *C. arietinum*, non-vernalized seeds were additionally sown. For each of the growing conditions (with or without vernalization), 6–8 seeds of each accession were planted at a time. The experiment was repeated twice to ensure a sufficient amount of samples for each accession.

We collected plant material from the following tissue types: (1) leaves before flowering initiation (leaves BF); (2) leaves after flowering initiation (leaves AF); and (3) flower buds at the initial stages of their formation (FB1 and FB2) [51,82]. The samples were collected during the daytime between 1 p.m. and 3 p.m. Leaves BF were harvested 15 days after sowing [82,83] by collecting a second uppermost leaf from each plant.

For each accession, tissue type, and condition, biological material was harvested from three plants, resulting in three independent biological replicates. The samples were placed into RNA later-stabilizing reagent (Thermo Fisher) at +4 °C and then stored at −20 °C.

In total, 144 samples with a sufficient amount of plant material were used for further RNA extraction.

### 4.2. RNA Extraction and Library Preparation

RNA was extracted using RNeasy Mini Kit (Qiagen). For each extraction, we took about 50 mg of plant material. RNA concentration was measured using a Qubit RNA BR Assay kit (Invitrogen, Carlsbad, CA, USA) and Qubit 2.0 fluorometer (Invitrogen). RNA quality was assessed by capillary electrophoresis on a Bio-analyzer 2100 (Agilent Technologies, Santa Clara, CA, USA) using a chip and an RNA 6000 Pico reagent kit (Agilent Technologies). The degree of RNA degradation was determined according to the RNA integrity index (RIN) [84].

For library preparation, we used RNA samples with RIN higher than 6.5. Preparation of cDNA libraries was performed using commercial NEBNext Ultra II RNA kits (New England BioLabs, Ipswich, MA, USA) according to the manufacturer’s protocol. To increase the proportion of target transcripts, we applied an additional step of poly (A) + mRNA enrichment using oligo dT probes. This resulted in the efficient removal of rRNA. The cDNA samples were purified with Agencourt AMPure XP magnetic beads (Beckman Coulter, Indianapolis, IN, USA). The concentration of the resulting libraries was measured with an DNA BR Assay kit (Invitrogen) and a Qubit fluorometer. The quality of the libraries was assessed by capillary electrophoresis using a High Sensitivity DNA reagent kit (Agilent Technologies). When a peak corresponding to the adapter dimers was detected in the library, additional purification was performed using magnetic beads.

### 4.3. Illumina Sequencing and Gene Expression Quantification

The libraries were sequenced using HiSeq4000 (Illumina) with a read length of 75 nucleotides in paired-end mode.

The sequencing reads were trimmed and filtered to remove low quality bases with AfterQC software [85] version 0.9.6. The average fraction of filtered out bases was 9.36%. Expression quantification was reformed using the kallisto program, version 0.46.1 [86], and the coding sequences of the *C. arietinum* genome, version 1.0 [87]. The average expression (tpm) was 3584.23.

### 4.4. Search for Orthologs of the *Arabidopsis* Flowering Time Genes

Flowering signaling pathways have been well studied in *Arabidopsis thaliana*, and are summarized in the interactive database of flowering time gene networks FLOR-ID (http://www.phytosystems.ulg.ac.be/florid/, accessed on 22 January 2023). These networks include 306 genes, most of which are members of multigenic families [6].

Most orthologs of the *Arabidopsis* flowering genes are not annotated in the reference chickpea genome. Thus, the expression of these genes cannot be analyzed using standard annotation, and orthologs must be identified. The nucleotide sequences of the *A. thaliana* flowering time genes from the FLOR-ID database were downloaded from Ensembl plants, genome version TAIR10. Their orthologs in the *C. arietinum* genome (version 1.0) were found using the tblastx program and their coding sequences were compared between *A. thaliana* and *Cicer*. The filtering threshold for candidate selection by bit score was set to 100. Accession numbers of several key regulators not identified via homologue search were taken from the literature [50].

As a result, 278 sequences of *Cicer* genes highly homologous to *A. thaliana* flowering time genes were included in the analysis. The short names of *Arabidopsis* genes (“Alias” in Supplementary Table S1) were taken from the section “Detailed Gene Information” of the FLOR-ID database.

### 4.5. Analysis of Differential Gene Expression

Differential expression analysis was performed using the DESeq2 package, version 1.28.1 [88]. Significant DEGs had an adjusted *p*-value (padj) < 0.01 and log2 fold change values greater than 1 or less than −1. To evaluate sampling between biological replicates, we applied the variance stabilizing transformation (VST) function from the DESeq2 package and then performed principal component analysis (PCA) on the transformed data. An example PCA plot is shown in Supplementary Figure S1.

**Figure 8:**
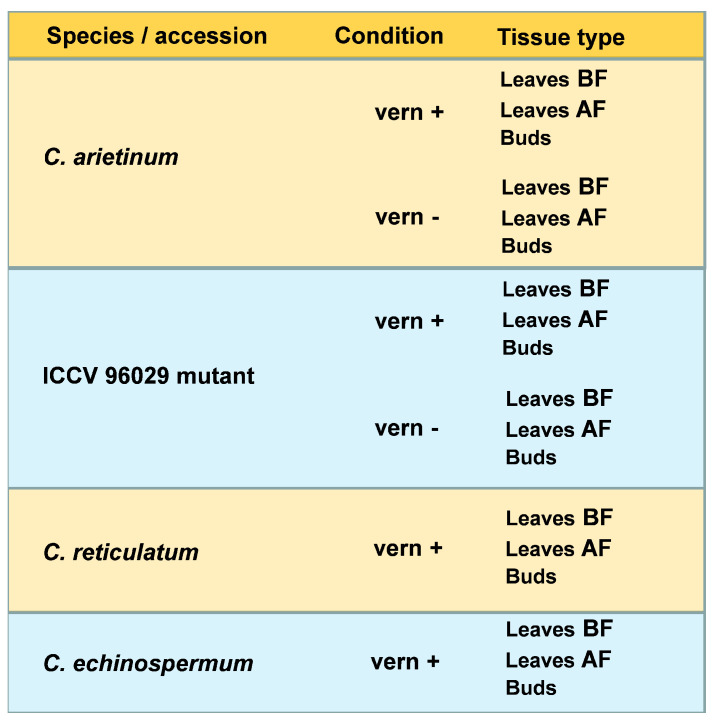
The dataset used for analysis of differential gene expression. ’leaves BF’—leaves before flowering initiation, ’leaves AF’—leaves after flowering initiation, ’buds’—early flower buds, ‘vern+’—after vernalization, ‘vern-’—without vernalization.

Gene expression did not differ significantly between the three *C. arietinum* accessions (ICC 16201, CDC Frontier, and Consul) or between individual accessions of *C. reticulatum* (Bari3 and Cudi 1022) or *C. echinospermum* (610380 and 610381); thus, we integrated RNAseq data for these accessions within each species. ICCV 96029, one of the world’s earliest chickpea cultivars, has been developed by the International Crop Research Institute for the semi-arid tropics (ICRISAT, India) [48,60]. It has recently been found to carry an 11-bp deletion in the first exon of the *ELF3* gene [35]. Here, we considered ICCV 96029 separately from the other *C. arietinum* varieties and referred to it as ‘mutant’. This resulted in four species/accessions being analyzed in this paper: *C. arietinum*, the ICCV 96029 mutant, *C. reticulatum*, and *C. echinospermum* (Figure 8).

DEGs were identified in the following pairwise comparisons: (1) between tissue types (species/accessions and conditions remained fixed): leaves BF—leaves AF; leaves AF—buds; buds—leaves BF; (2) between conditions (species/accessions and tissue types remained fixed): without vernalization—after vernalization; and (3) between species/accessions (conditions and tissue types remained fixed): *C. arietinum*—ICCV 96029 mutant; *C. arietinum*—*C. reticulatum*; *C. arietinum*—*C. echinospermum*, ICCV 96029 mutant—*C. reticulatum*; ICCV 96029 mutant—*C. echinospermum*; *C. reticulatum*—*C. echinospermum*.

We attributed chickpea orthologs to the following pathways according to the information from the FLOR-ID and TAIR databases [6,89]: photoperiodism, light perception, and signaling; vernalization and temperature; hormones, Gibberellin signaling, and metabolism; sugar pathway; general processes and autonomous pathway; development; and main targets of floral homeotic genes. We used the following short names of these pathways: (1) photoperiod and circadian clock; (2) vernalization and temperature; (3) hormone; (4) sugar; (5) autonomous; (6) development; and (7) main targets.

*A. thaliana* flowering time gene products are frequently involved in a variety of biological processes. For the sake of convenience, in our analysis each gene was attributed to one major pathway (Supplementary Table S1), with their additional roles discussed in the text.

### 4.6. Real-Time PCR Assay

The RNA concentrations in the samples were determined using a Qubit 4 fluorometer (Thermo Fisher, Waltham, MA, USA) and an RNA HS Assay Kit (Thermo Fisher). RNA integrity was assessed by electrophoresis using 1% agarose gel with GelRed^®^ Nucleic Acid Gel Stain (Biotium). The first complementary DNA (cDNA) strands were obtained by reverse transcription using an MMLV RT Kit (Evrogen, Moscow, Russia) according to the manufacturer’s protocol. cDNA concentration prior to quantitative PCR was measured using a P360 Nanophotometer (Implen, München, Germany). Negative controls (no enzyme) were used to monitor genomic DNA contamination.

**Figure A3:**
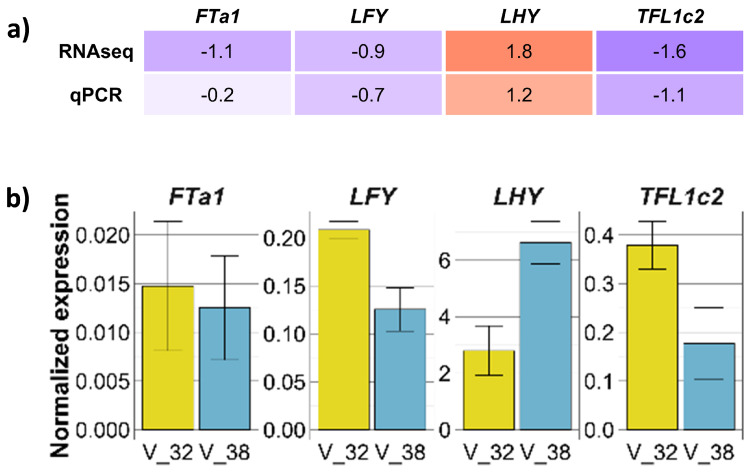
Application of quantitative real-time PCR for verification of transcriptomic data: (**a**) log2-transformed fold changes in the expression levels of indicated genes between two randomly chosen samples resulting from RNAseq and qPCR experiments and (**b**) the results of qPCR.

Oligonucleotide primers for qPCR were constructed using the BeaconDesigner software tool and synthesized by Evrogen Company (Supplementary Table S2). Quantitative PCR was performed using a CFX96 Touch Real-Time PCR Detection System with three replicates for each point. The expression levels of target genes were normalized to the expression of *ACT1* or *EF1α* (Figure A3b).

## Data Availability

Not applicable.

## Supplementary Materials

The following supporting information can be downloaded at: https://www.mdpi.com/article/10.3390/ijms24032692/s1.

## Appendix A

### Appendix A.1. The Number of Copies of Flowering Time Genes in Cicer Dataset

Of the 278 analyzed *Cicer* orthologs of the *Arabidopsis* flowering time genes, 48 genes had two or more copies in the genome (Supplementary Table S1).

We detected multiple duplications, including a duplication in the *CRY2* gene ortholog, which is common to all legumes [34,90]. *CRY2* encodes the blue light receptor and regulates photoperiodic flowering in *Arabidopsis*. In chickpea, the *Ca_12132* and *Ca_23164* genes are the orthologs of *Arabidopsis CRY2* gene (*AT1G04400*; see Supplementary Table S1).

In addition to *CRY2*, several other orthologs of the *Arabidopsis* genes attributed to the ‘photoperiod/circadian clock’ pathway were present in more than one copy: *PHYA*, *SPA1-RELATED 1 (SPA1)*, *CIB1*, and *FLAVIN-BINDING, KELCH REPEAT, F BOX 1 (FKF1)* had two copies, while *CPK6* and *CPK33* [91] had nine and eight copies, respectively. The chickpea orthologs of the *TPL* and *SUS4* genes, which in *Arabidopsis* belong to the ‘main targets’ and ’sugar’ pathways, respectively, were present in seven copies (Supplementary Table S1).

With respect to *Cicer FT* and *TFL1* orthologs, we did not detect differential expression of the *FTa2*, *FTc*, or *TFL1a* genes in our dataset, which resulted in three *FT* genes (*FTa1, FTa3*, and *FTb*) and four *TFL1* genes (*TFL1b, TFL1c1, TFL1c2*, and *TFL1c3*).

We found fourteen copies of the *FT-INTERACTING PROTEIN 1 (FTIP1)* ortholog, which in *Arabidopsis* plays an important role in the FT protein transport (Supplementary Table S1).
