# Supplementary material for: Transcriptomic Analysis of Flowering Time Genes in Cultivated Chickpea and Wild Cicer
Source: Int J Mol Sci. 2023 Jan 31;24(3):2692. doi: 10.3390/ijms24032692 (PMC9916832; doi:10.3390/ijms24032692)
Supplement: Supplementary file 1 [file ijms-24-02692-s001.zip › Supplementary Figure S1.pdf]

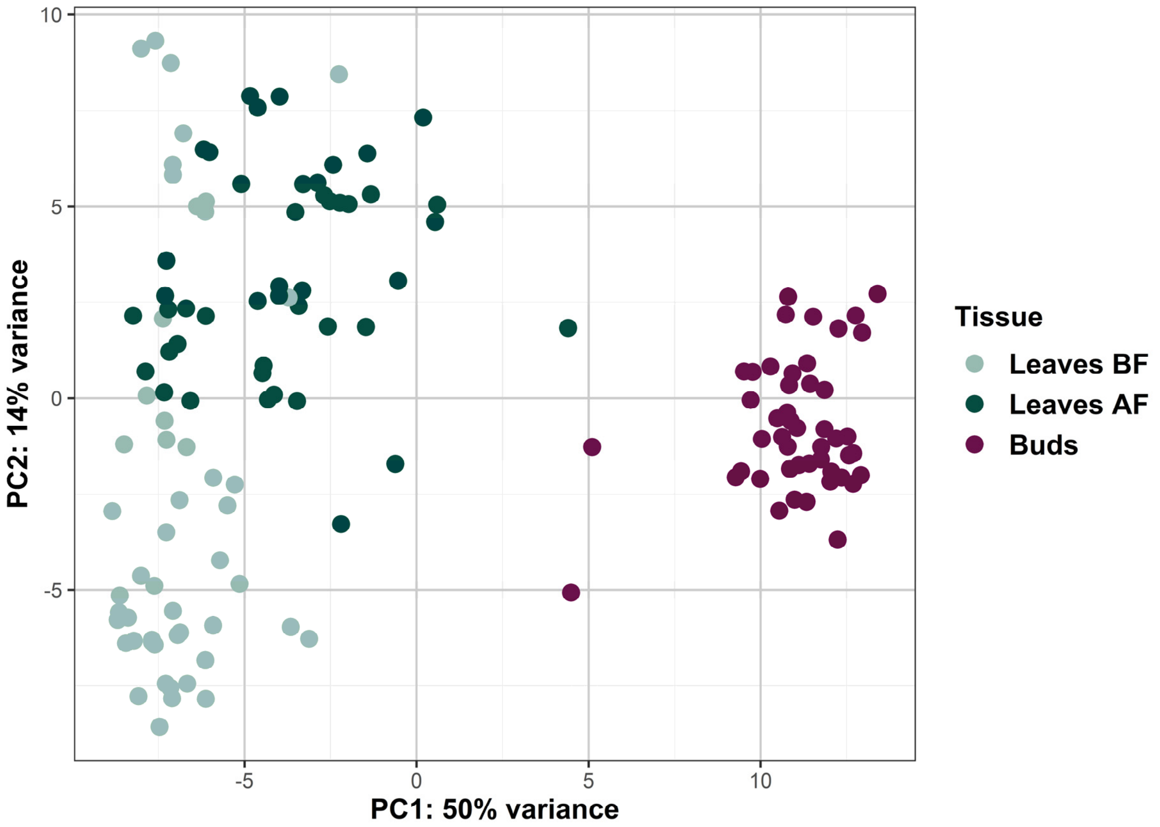

Supplementary Figure S1: Principal component analysis (PCA) plot which resolves samples 558 according to the tissue type.
