# Supplementary material for: Transcriptomic Analysis of Flowering Time Genes in Cultivated Chickpea and Wild Cicer
Source: Int J Mol Sci. 2023 Jan 31;24(3):2692. doi: 10.3390/ijms24032692 (PMC9916832; doi:10.3390/ijms24032692)
Supplement: Supplementary file 1 [file ijms-24-02692-s001.zip › Supplementary Table S2.pdf]

Supplementary Table 2: Primer Details

CA_ID	Name	Primer sequence (5' to 3')	Tm
Ca_01365	*LHY*	F: GACTGATGCGTTGGAGAA	54
		R: ACACCTGCCTGGAGAATA	54
Ca_01386	*LFY*	F: GTTGACCGTGATGAGAAT	52
		R: GGCATTGTTCGTAGAGAT	52
Ca_TFL1c2	*TFL1c*	F: CCAGGCACAACAGATT	48
		R: AAGGACGAACACATACC	50
Ca_FTa1	*FTa1*	F: GGCGTAGGAGTGTAAGAA	54
		R: TGATTGGCAACTTGAGAAG	54
